# A genetic labeling system to study dendritic spine development in zebrafish models of neurodevelopmental disorders

**DOI:** 10.1242/dmm.049507

**Published:** 2022-08-19

**Authors:** Elisabeth C. DeMarco, George R. Stoner, Estuardo Robles

**Affiliations:** Department of Biological Sciences and Purdue Institute for Integrative Neuroscience, Purdue University, West Lafayette, IN 47907, USA

**Keywords:** Neuronal development, Optic tectum, Synaptogenesis

## Abstract

Dendritic spines are the principal site of excitatory synapse formation in the human brain. Several neurodevelopmental disorders cause spines to develop abnormally, resulting in altered spine number and morphology. Although spine development has been thoroughly characterized in the mammalian brain, spines are not unique to mammals. We have developed a genetic system in zebrafish to enable high-resolution *in vivo* imaging of spine dynamics during larval development. Although spiny neurons are rare in the larval zebrafish, pyramidal neurons (PyrNs) of the zebrafish tectum form an apical dendrite containing a dense array of dendritic spines. To characterize dendritic spine development, we performed mosaic genetic labeling of individual PyrNs labeled by an *id2b:gal4* transgene. Our findings identify a developmental period during which PyrN dendrite growth is concurrent with spine formation. Throughout this period, motile, transient filopodia gradually transform into stable spines containing postsynaptic specializations. The utility of this system to study neurodevelopmental disorders was validated by examining spine development in *fmr1* mutant zebrafish, a model of fragile X syndrome. PyrNs in *fmr1* mutants exhibited pronounced defects in dendrite growth and spine stabilization. Taken together, these findings establish a genetic labeling system to study dendritic spine development in larval zebrafish. In the future, this system could be combined with high-throughput screening approaches to identify genes and drug targets that regulate spine formation.

## Abstract (precis)

**Summary:** We have developed a genetic labeling system in zebrafish to enable high-resolution *in vivo* imaging of dendritic spine dynamics during larval development.

## INTRODUCTION

Dendritic spines are the principal site of excitatory synapse formation in the human brain (Nimchinsky et al., 2002; Yuste, 2013). The developmental processes leading to spine formation have been well characterized in the mammalian brain. Following the establishment of neuronal polarity, dendrites grow and branch to form an arbor defining the neuron's synaptic input field (Ledda and Paratcha, 2017). Following this phase, the arbor is remodeled via activity-dependent addition or removal of branches. Spine development is then initiated by formation of filopodia from the dendritic shaft (Ziv and Smith, 1996). Filopodia are motile, antenna-like protrusions that sample the environment for extracellular cues and potential synaptic partners. Contact with a synaptic partner leads to local recruitment of postsynaptic density (PSD) scaffolding proteins such as PSD95 (encoded by *dlg4b*), a membrane-associated guanylate kinase (MAGUK) with a role in localizing glutamate receptors to PSDs (Chen et al., 2015). Formation of a synapse stabilizes the filopodium and initiates its transformation into a mature spine with an enlarged head containing a PSD (Harris, 2020). Many lines of evidence indicate that this process is disrupted in several neurodevelopmental disorders, including autism spectrum disorders (ASDs). For example, in mouse *fmr1* knockout models of fragile X syndrome, immature spine morphologies have been described in several brain areas, including the cortex (Comery et al., 1997; Cruz-Martín et al., 2010; Nimchinsky et al., 2001; Pan et al., 2010), hippocampus (Grossman et al., 2006), cerebellum (Koekkoek et al., 2005) and amygdala (Qin et al., 2011). These studies suggest that impaired spine maturation is a hallmark morphological defect in fragile X syndrome. However, the cellular pathways that control spine development downstream of *fmr1* and other ASD risk genes are not well understood. The larval zebrafish is a prominent model for high-throughput drug screening (MacRae and Peterson, 2015), as well as forward and reverse genetic screening (Muto et al., 2005; Shah et al., 2015). Therefore, establishing a system to analyze *in vivo* spine development in zebrafish larvae holds the promise of identifying novel cellular signaling pathways that are disrupted in fragile X syndrome and other ASDs.

Dendritic spines are not unique to the mammalian brain – they are also found in cold-blooded vertebrates and invertebrates. Remarkably, structures resembling dendritic spines are also common in neurons in planarians, the simplest living animals with a bilateral body plan (Sarnat and Netsky, 1985). Although rare in insects, honeybee Kenyon cells in the mushroom body have dendrites that are densely decorated with spine-like protrusions (Groh and Rössler, 2020). Kenyon-cell spine densities remain constant during development, yet exhibit a shift towards shorter and thicker morphologies (Farris et al., 2001). In cold-blooded vertebrates, dendritic spines have been described in *Xenopus* olfactory bulb interneurons (Zhang et al., 2016) and in tectal interneurons of the jewel fish (Coss and Globus, 1979). However, the aforementioned systems lack genetic methods to consistently label these populations of spiny neurons. In contrast, zebrafish are genetically tractable, enabling generation of transgenics that label specific tissues and cell types. Zebrafish are also optically transparent during the larval stages of development, making *in vivo* imaging of neuronal dendrites less time consuming compared to rodent models, which require surgical craniotomy. A genetic system to study dendritic spines in larval zebrafish would enable high-throughput analysis of dendritic spine dynamics *in vivo*.

One neuron type in the adult teleost tectum known to form dendritic spines is the type I/pyramidal neuron (PyrN) (Folgueira et al., 2020; Ito and Kishida, 2004; Meek, 1990; Vanegas et al., 1974; Xue et al., 2003). We recently described an *id2b:gal4* transgenic that labels PyrNs in the larval zebrafish tectum (DeMarco et al., 2019). A subsequent study used sparse genetic labeling to demonstrate that PyrNs, despite forming small synaptic territories, are densely innervated, with as many as 100 postsynaptic specializations (Demarco et al., 2021). The presence of dendritic spines along the apical dendrite facilitates high input density in the PyrN (Demarco et al., 2021; Folgueira et al., 2020; Laufer and Vanegas, 1974) and creates a distinct subcellular compartment for each synaptic input. The ability to consistently label a genetically defined population of spiny neurons will allow us to exploit the strengths of the zebrafish model system to examine the mechanisms of spine formation in neurodevelopmental disease states. A necessary first step towards this goal is to define the dynamics of spine formation and maturation in the wild-type condition. Therefore, we set out to characterize the process of spine maturation in the larval zebrafish tectum with the following aims: (1) to identify an appropriate developmental window to study spine development; (2) to monitor protrusion dynamics during the filopodia-to-spine transition; (3) to define the relationship between PSD95 accumulation and spine development; and (4) to determine whether PyrN spine maturation is disrupted in *fmr1* mutant larvae.

Our findings identified a developmental window in early larval development between 4 and 11 days during which PyrN dendrite remodeling (branch addition/elimination) was concurrent with spine formation. Throughout this period, motile, transient filopodia were gradually replaced by short, stable spines. During this transition, the postsynaptic protein PSD95 localized to most spines, yet its accumulation was a weak predictor of spine stability. Structural imaging of PyrN apical dendrites in *fmr1* mutants revealed smaller dendrite arbors with reduced spine densities. Morphologically, these spines also had thinner heads compared to those in wild-type larvae. Time-lapse imaging revealed that these spines exhibited reduced stability. These spine defects are consistent with previous findings in mammals and establish the *id2b:gal4* transgenic as a valuable tool to study spine development in a genetically tractable and optically transparent vertebrate.

## RESULTS

### Tectal PyrNs contain spiny apical dendrites

**Fig. 1.**
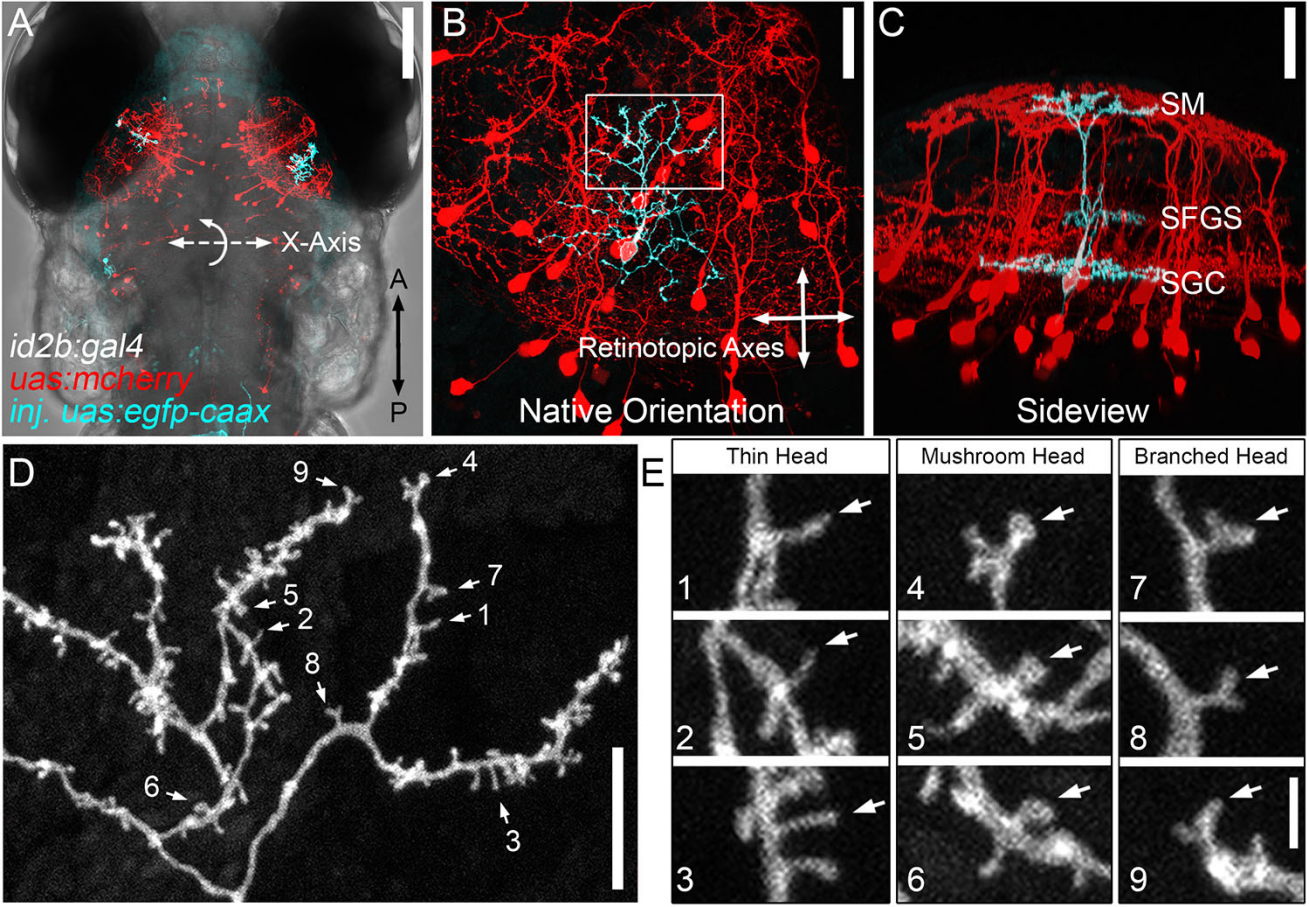
**Dendritic spines on PyrN apical dendrites.** (A) Dorsal view, whole-brain confocal image volume of an 8 dpf double transgenic *Tg(id2b:gal4,uas-e1b:ntr-mCherry)* larva injected at the embryo stage with *uas:egfp-caax* plasmid to generate sparse labeling. Note the single EGFP-labeled PyrN in each tectal lobe. (B) Higher magnification of the maximum projection of neurons labeled in the right tectal lobe of the larva in A. Projection is shown from the dorsal view, with 0° rotation. Note that this view was used for subsequent measurements of dendrite area along the retinotopic axes. (C) Maximum projection of same neuron rotated −50° about the *x*-axis, to yield an orientation parallel to the tectal layers. Note the clearly stratified neurite morphology with arbors in the SM, SFGS and SGC layers of the tectal neuropil. (D) High magnification view of a subvolume of the SM-targeted dendrite of PyrN in B,C as indicated by the box in B. Note the branched arbor decorated with multiple short protrusions. Numbered arrows indicate spines selected for higher magnification views. (E) 3× magnified views of nine dendritic spines indicated by arrows in D. Note the presence of different types of spine heads: thin, mushroom and branched. Images are representative of 20 PyrNs. Scale bars: 100 µm (A); 25 µm (B,C); 10 µm (D); 1.65 µm (E).

We previously characterized PyrNs as the tectal neuron most frequently labeled in the *id2b:gal4* transgenic (DeMarco et al., 2019). To confirm the utility of the *id2b:gal4* transgenic to visualize spine formation in PyrNs, we performed mosaic genetic labeling via injection of *Tg*(*id2b:gal4,uas-e1b:ntr-mcherry*) transgenic embryos with plasmids encoding fluorescent reporters under the control of an upstream activator sequence (UAS) enhancer region. Preliminary tests using cytosolic fluorescent protein expression were suboptimal due to large differences in the fluorescence signal between dendrites and their spines, likely due to the large volume difference between these compartments (Fig. S1). In contrast, a membrane-targeted EGFP (*uas:egfp-caax*) resulted in comparable fluorescence signal intensity in both dendrites and spines (Fig. 1A-D). This enhanced spine labeling permitted the use of reduced laser powers for confocal microscopy, which, in turn, enabled time-lapse imaging over several hours and repeated time-lapse imaging of the same dendrite over several days of development.

Typically, injection of 200 two- to four-cell-stage embryos yielded several larvae with strong, sparse labeling of isolated neurons in the tectum (Fig. 1A). The *id2b:gal4* transgenic labeled three tectal neuron types: PyrNs (73.5%), torus longitudinalis projecting neurons (11.8%) and tegmentum projecting neurons (14.7%) (DeMarco et al., 2019). These three neuron types can be consistently distinguished morphologically by 4 days post fertilization (dpf). PyrNs were primarily identified by their dendritic stratification pattern, with three arbors formed in different synaptic layers of the tectal neuropil (Fig. 1C). The apical dendrite is formed in the superficial-most stratum marginalis (SM) layer and receives excitatory input from the torus longitudinalis (TL), a second-order visual area in fish (Fig. 1C) (Folgueira et al., 2020; Northmore, 2017). The medial dendrite is formed in the stratum fibrosum grisealis (SFGS) layer and has been shown to receive direct inputs from retinal axons (Laufer and Vanegas, 1974). The basal dendrite situated in the stratum grisealis centralis (SGC) is a mixed neurite arbor containing both axonal and dendritic branches (Demarco et al., 2021). In every PyrN imaged, the developing neuron resembled its mature, tristratified form at 4 dpf (*n*=9 PyrNs from eight larvae). High-resolution imaging of PyrNs labeled by the *id2b:gal4* transgene confirmed numerous spine-like protrusions on the apical dendrite at 8 dpf (Fig. 1D). Structurally, these ranged from thin, filopodia-like protrusions to ones with mushroom-shaped heads (Fig. 1E). Additionally, we observed branched spines with two distinct heads (Fig. 1E). Spines with mushroom or branched heads typically exhibited lengths in the range of 0.5 to 2 µm, whereas thin filopodia-like protrusions were as long as 5 µm. In general, the shapes and dimensions of larval PyrN spines were similar to those in mammals (Sheng and Kim, 2011) and adult zebrafish (Bayés et al., 2017).

### PyrN dendrite stratification precedes dendritic arbor stabilization

**Fig. 2.**
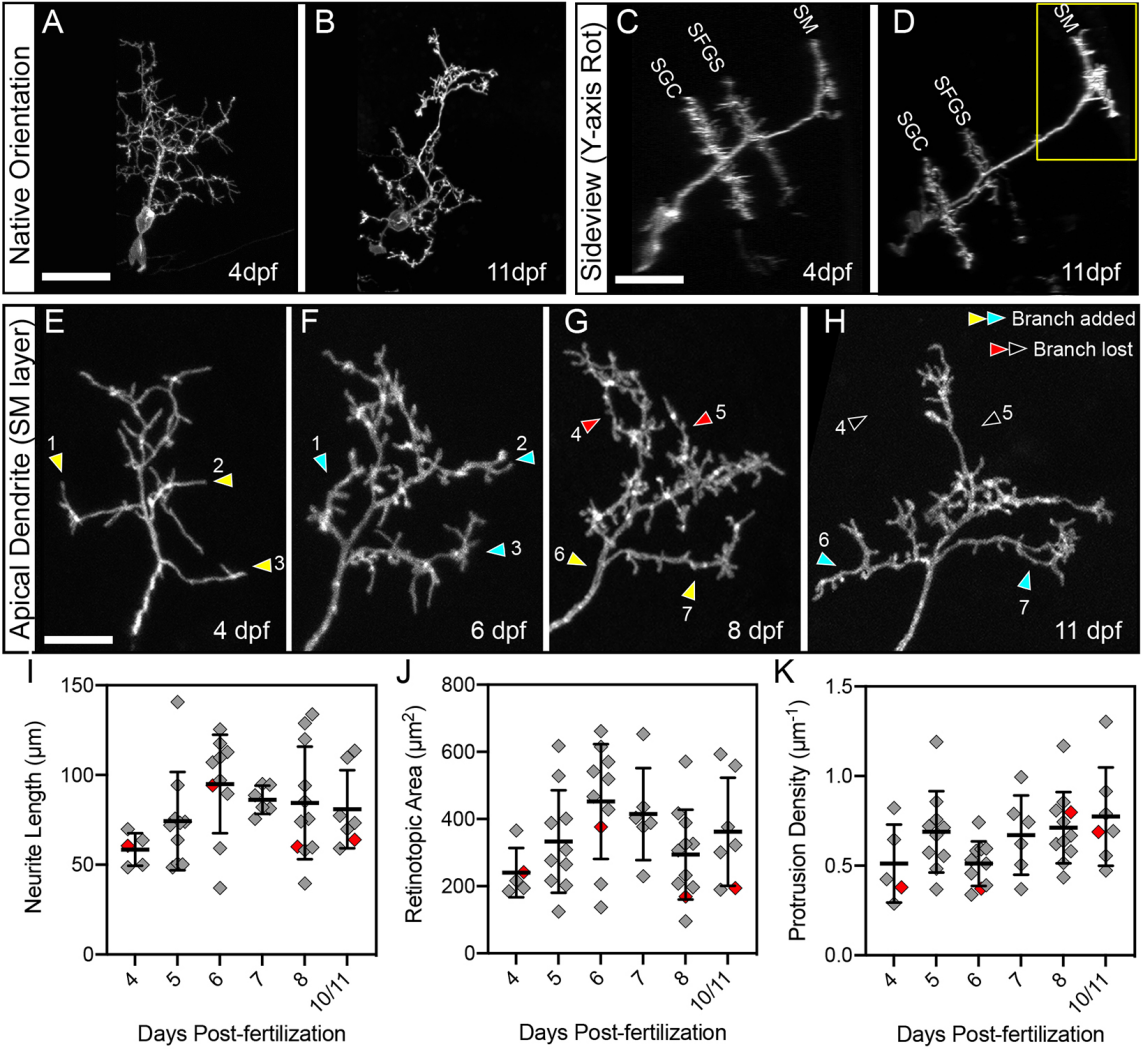
**Multi-day imaging of dendrite development and spine formation.** (A,B) Native-orientation (dorsal view) maximum-projection images of the same PyrN at 4 dpf and 11 dpf. (C,D) Rotated sideview projections of the neuron in A,B. Three distinct dendritic stratifications are visible at each timepoint. Also note that the total length of the main dendritic process increases, most likely due to thickening of the tectal neuropil during this developmental period. (E-H) Higher magnification views of the SM-targeted apical dendrite of PyrN in A-D (region indicated by the yellow box in D). Sites where new branches are added are indicated by yellow arrowheads (before branch addition) and cyan arrowheads (after branch addition). Sites where branches were retracted are indicated by red arrowheads (before branch retraction) and open arrowheads (after branch retraction). Note that branches are added and retracted throughout this time window. Also note that from 4 to 11 dpf there is a gradual loss of long, filopodial protrusions and an increase in short, spine-like protrusions. (I) Neurite length measurements of PyrN SM dendrites between 4 and 10/11 dpf. Due to reduced survival at the 10 and 11 dpf timepoints, these groups were combined into one bin. (J) Retinotopic area measurements of PyrN SM dendrites between 4 and 10/11 dpf. (K) Spine density measurements of PyrN SM dendrites between 4 and 10/11 dpf. Number of neurons and larvae (indicated in parentheses) analyzed for each timepoint: 5 (5), 10 (10), 10 (10), 6 (6), 11 (11) and 7 (7). All graphs depict mean±s.d. Red diamonds indicate measurements obtained from PyrN dendrite in E-H. One-way ANOVA with Tukey's multiple comparisons test was used to determine significance, *P*-values for all pairwise comparisons in I, J and K were >0.05. Scale bars: 20 µm (A-D); 5 µm (E-H).

To determine the order in which the characteristic morphological features of PyrNs are established, we conducted multi-day imaging of single PyrNs labeled with EGFP-caax. Two hallmark structural features of PyrNs are a tristratified dendrite and an apical dendritic arbor containing a dense constellation of spines. If spine formation does serve an instructive role in guiding dendrite stratification, we reasoned that these two processes should be concurrent. In three larvae, we were able to perform longitudinal, multi-day imaging of the same PyrN at 4, 6, 8 and 11 dpf. Confocal image volumes acquired at 4 dpf revealed PyrNs with highly branched dendritic arbors (Fig. 2A). Between 4 and 11 dpf, the arbor underwent structural rearrangements and appeared to have fewer fine branches (Fig. 2B). Sideview rotations of these image volumes enabled visualization of the three characteristic PyrN dendrite stratifications (Fig. 2C,D). In every neuron examined, the stratification pattern remained constant from 4 to 11 dpf. During the same period, the vertically oriented primary dendrite branch increased in length, owing to increasing thickness of the tectal neuropil (Fig. 2D). Although the stratification pattern remained largely unchanged, arbors did undergo structural rearrangements along the retinotopic axes (orthogonal to stratification layers). The most notable rearrangement in the PyrN shown in Fig. 2 was an increase in arbor size between 4 and 6 dpf (Fig. 2E,F). Examination of the apical dendrite revealed that it had a highly filopodial morphology at 4 dpf (Fig. 2E), whereas protrusions with spine-like shapes predominated at later timepoints (>6 dpf; Fig. 2F-H). This confirms that the PyrN stratification pattern is established early and remains stable throughout the period of larval development examined (4-11 dpf). PyrN dendrite stratification can be genetically specified and independent of activity-dependent refinements controlling arborization along the retinotopic axes. This would be consistent with previous findings that synaptic layering in the tectum is ‘hardwired’ and unaffected by blockade of neuronal activity or neurotransmitter release (Nevin et al., 2008).

### PyrN apical dendrite arborization is concurrent with the filopodia-spine transition

Synaptotropic models of neurite growth propose that synapse formation guides dendrite arborization (Cline and Haas, 2008; Niell, 2006). As spines are likely the main site of synapse formation on PyrN apical dendrites, this would require concurrent dendrite growth and spine formation. To determine the time-course of apical arbor stabilization relative to the filopodia-spine transition, we examined image volumes containing the apical dendrite (boxed region in Fig. 2D) over multiple days of development. This allowed us to monitor dendritic branches gained and lost over time (see Fig. 1B). Fig. 2E-H shows the structural development of a single PyrN apical dendrite at 4, 6, 8 and 11 dpf. At 4 dpf, this PyrN had a compact apical dendrite containing several branches and many filopodial protrusions (Fig. 2E). Between 4 and 6 dpf, this arbor underwent an increase in size that was driven by the addition of three new branches (yellow and cyan arrowheads in Fig. 2E,F). By 6 dpf, this dendrite had a mix of filopodia-like protrusions and short, spine-like protrusions. From 6 to 8 dpf, no branches were gained or lost and the total area was similar (Fig. 2F,G); however, the majority of protrusions were short and spine-like at this timepoint. Between 8 and 11 dpf, the arbor gained two branches (yellow and cyan arrowheads in Fig. 2G,H) and lost two branches (red and open arrowheads in Fig. 2G,H). Throughout these changes in shape, the dendrite maintained a similar density of spine-like protrusions (Fig. 2G,H). This is reminiscent of previous findings in aspiny tectal neurons, in which dendrite length and synapse number increase between 3 and 7 dpf, but then remain constant between 7 and 10 dpf (Niell et al., 2004).

To quantitatively assess PyrN apical dendrite growth during the filopodia-spine transition, we analyzed three morphological parameters between 4 and 11 dpf: total neurite length, retinotopic area and protrusion density. Three-dimensional (3D) neurite lengths were calculated by generating skeletonized tracings of the dendrite. The retinotopic area was calculated using a convex polygon connecting the outermost branch tips of each arbor along the retinotopic axes (see Fig. 1B). The protrusion density was calculated by manually annotating protrusion locations on maximum projections of dendrite image volumes. Neurite length gradually increased between 4 and 6 dpf, followed by a slight decrease between 6 and 10 dpf (Fig. 2I). A similar pattern was observed when examining the retinotopic area of PyrN apical dendrites area between 4 and 11 dpf. However, these trends were not statistically significant (Fig. 2I,J; one-way ANOVA with Tukey's multiple comparisons test). Although their morphology appeared to change over time, the dendritic protrusion density remained relatively constant between 5 and 11 dpf (5 versus 6, 7, 8 or 10/11 dpf; all *P*-values>0.418; Fig. 2K). This suggests that apical dendrites are simultaneously sampling a large number of potential presynaptic partners during the early stages of arborization.

**Fig. 3.**
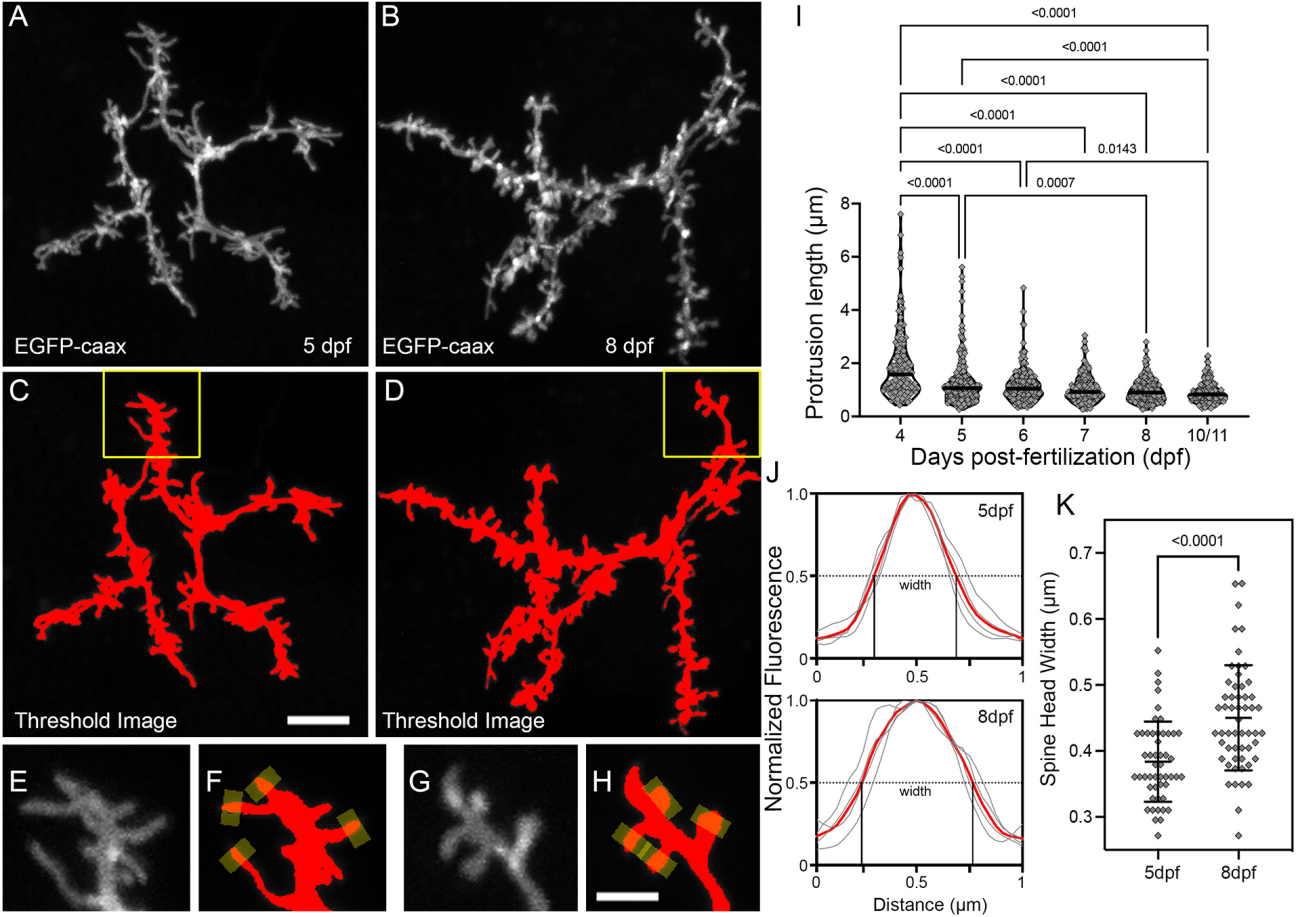
**Morphological changes associated with the filopodia-spine transition.** (A,B) Maximum projections of apical dendrite subvolumes acquired from a 5 dpf PyrN (A) and an 8 dpf PyrN (B). (C,D) Examples of images in A,B with a low threshold mask applied. (E-H) Magnified views of dendrite subregions indicated by yellow boxes in C and D. Grayscale (E,G) and thresholded (F,H) images are presented for each region. Yellow rectangles at spine tips in F,H indicate 0.5 µm-wide line selections used to generate fluorescence intensity plots across each spine head. (I) Quantification of protrusion lengths for PyrN apical dendrites at 4, 5, 6, 7, 8 and 10/11 dpf. Note gradual decrease in protrusions with lengths greater than 2 µm. The median for each group is indicated by a horizontal line. Number of spines and neurons (indicated in parentheses) analyzed at each timepoint: 123 (6), 225 (7), 214 (7), 137 (5), 187 (5) and 157 (4). One-way ANOVA with Tukey's multiple comparisons test was used to determine significance. All significant differences (*P*<0.05) are shown on graph. (J) Spine head fluorescence intensity plots for measuring spine head widths. Thin gray traces are for the four spines indicated in F and H. Width was calculated by measuring the width at half-maximum for each trace. The means of these widths for each set is indicated by red traces. (K) Comparison of spine head widths at 5 and 8 dpf. Number of spines and neurons (indicated in parentheses) analyzed at 5 and 8 dpf: 50 (4) and 55 (4). Data are presented as mean±s.d. Significance was determined using a two-tailed unpaired *t*-test. Scale bars: 5 µm (A-D); 2 µm (E-H).

To quantify protrusion maturity during this developmental period, we measured two morphological metrics: protrusion length and head width. To minimize the possibility of underrepresenting thinner or dimmer protrusions, we performed these analyses on images generated using a permissive threshold mask, so that all spines were filled with equal pixel intensities (Fig. 3A-D). These analyses included isolated spines located anywhere on the dendritic arbor. In total, length measurements were obtained from 1043 protrusions (>120 at each timepoint, >5 neurons at each timepoint). From 4 to 11 dpf, the average length of protrusions gradually decreased (Fig. 3I), driven by a reduction in the number of protrusions that were >2 µm in length. To examine spine head width, we acquired image volumes from 5 and 8 dpf PyrNs at increased resolution (pixel sizes between 0.03 and 0.08 µm) by using a higher numerical aperture objective and increasing image size. The early timepoint of 5 dpf was chosen because these PyrN dendrites exhibited a high density of filopodia-like protrusions at this stage (Fig. 3A,C,I). The late timepoint of 8 dpf was chosen as the majority of protrusions at this stage were spine-like and <2 µm in length (Fig. 3B,D,I). Head widths were measured by obtaining intensity profiles from regions spanning the distal 0.5 µm of each protrusion (Fig. 3E-H). From these traces, we analyzed the width of each curve at half-maximum to generate width measurements for >50 spines at each age (Fig. 3J). Although this approach might slightly underestimate the width, the values obtained were similar to the spine head widths observed in the adult zebrafish tectum (Bayés et al., 2017). Despite a range of widths observed at both timepoints, we did detect a significant increase in protrusion head width from 5 to 8 dpf (*P*<0.001, unpaired two-tailed *t*-test; Fig. 3K), reflecting an increase in spine-like morphologies. Concurrent dendrite arborization and the filopodia-spine transition is consistent with a model in which spine formation guides dendritic growth and branching.

### Changes in spine morphology correlate with increased stability

**Fig. 4.**
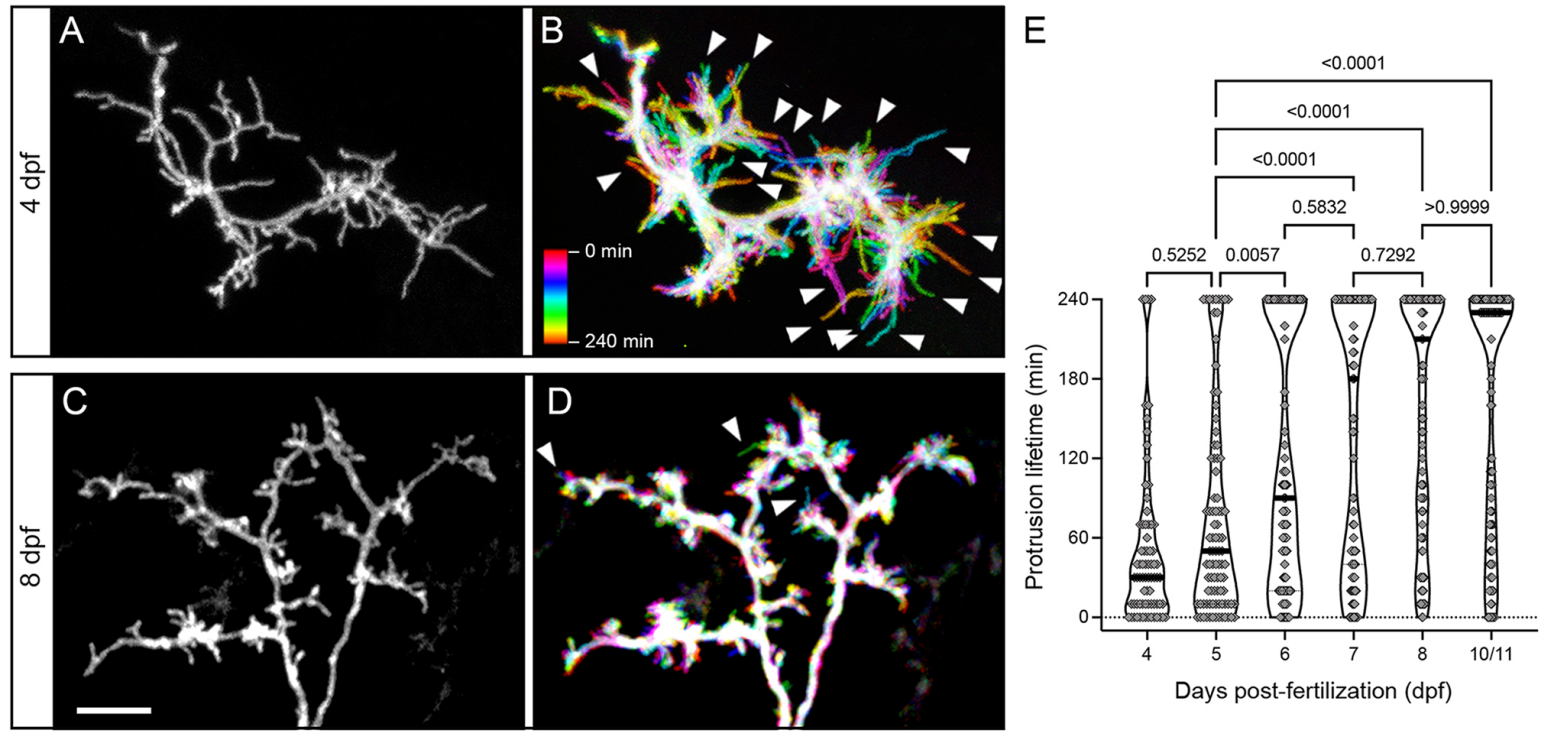
**Developmental changes in protrusion stability.** (A) High magnification view of the SM-targeted apical dendrite of a 4 dpf PyrN. Note the abundance of long, filopodial protrusions. (B) Temporal color-coded image of the dendrite in A during a 4 h time-lapse recording with image stacks acquired every 10 min. The color scale indicates which image corresponds to each timepoint. Note the prevalence of motile protrusions with a single-color label, indicating that they were present at that position during a single acquisition (arrowheads). (C) High magnification view of the SM-targeted apical dendrite of an 8 dpf PyrN. Note the abundance of short, spine-like protrusions. (D) Temporal color-coded image of the dendrite in C during a 4 h time-lapse recording with image volumes acquired every 10 min. Note the relatively small number of filopodia-like motile protrusions generated (arrowheads). (E) Comparison of protrusion lifetimes at 4, 5, 6, 7, 8 and 10/11 dpf. Note the gradual increase in stable protrusions (240 min lifetimes) and decrease in transient spines lasting less than 120 min. The horizontal lines on the violin plots indicate median values for each group. One-way ANOVA with Tukey's multiple comparisons test was used to determine significance. Significant differences not shown on graph: 4 dpf versus 6 dpf, *P*<0.0001; 4 dpf versus 7 dpf, *P*<0.0001; 4 dpf versus 8 dpf, *P*<0.0001; 4 dpf versus 10/11 dpf, *P*<0.0001; 6 dpf versus 8 dpf, *P*=0.015; and 6 dpf versus 10/11 dpf, *P*=0.0154. Number of spines and neurons (indicated in parentheses) analyzed at each timepoint: 73 (4), 91 (5), 99 (4), 83 (4), 123 (4) and 110 (4). Scale bar: 5 µm.

Our first indication that dendritic protrusions become more stable during larval development came from time-lapse recordings of neurons used for multi-day structural imaging (Fig. 2). At 4 dpf, arbors contained motile filopodia that were dynamically extending and retracting, whereas at 8 dpf, most protrusions were spine-like and present for several hours (Movie 1). However, even these stable spines present for the entire 4 h recording constantly underwent small changes in shape and length (typically ≤1 µm). To qualitatively demonstrate the difference in the number of motile protrusions, we generated color-coded temporal projections from dendritic time-lapse recordings (Fig. 4A-D). These images were made by assigning a unique color to each timepoint and combining these single-color images into a maximum projection image. In the resulting image, motile and short-lived protrusions are labeled with a single color, whereas stable regions appear white (composite of all colors in the lookup table). Although 4 dpf dendrites contained many motile and short-lived protrusions, 8 dpf dendrites contained very few (arrowheads in Fig. 4B,D). To quantitatively assess the stability of dendritic spines, we directly measured protrusion lifetimes from 4 h time-lapse recordings collected at 4, 5, 6, 7, 8 and 10/11 dpf (*n*=5 neurons and >100 protrusions per timepoint; Fig. 4E). At 4 and 5 dpf, stages when PyrN apical dendrites have many filopodia-like protrusions, very few protrusions had lifetimes ≥230 min (5.5% and 12.1%, respectively; *n*=73 and 91; Fig. 4E). Conversely, at 7, 8 and 10/11 dpf, close to half the protrusions had lifetimes ≥230 min (40.1%, 48% and 55.5%, respectively; *n*=83, 123 and 110; Fig. 4E). The 6 dpf timepoint represented an intermediate stage at which 34.3% of protrusions were stable. Statistical testing confirmed a significant difference between early (4-5 dpf) and late (7, 8 and 10/11 dpf) timepoints (*P*<0.0001 for all pairwise comparisons between early and late timepoints, one-way ANOVA with Tukey's multiple comparisons test). This pattern is the opposite of what we observed for protrusion lengths, which incrementally decreased during this period (Fig. 3I). Therefore, the shift from filopodial to spine-like morphologies coincides with an increase in protrusion stability, likely due to stable synaptic contact.

### PSD95-EGFP accumulation is weakly correlated with spine stability

**Fig. 5.**
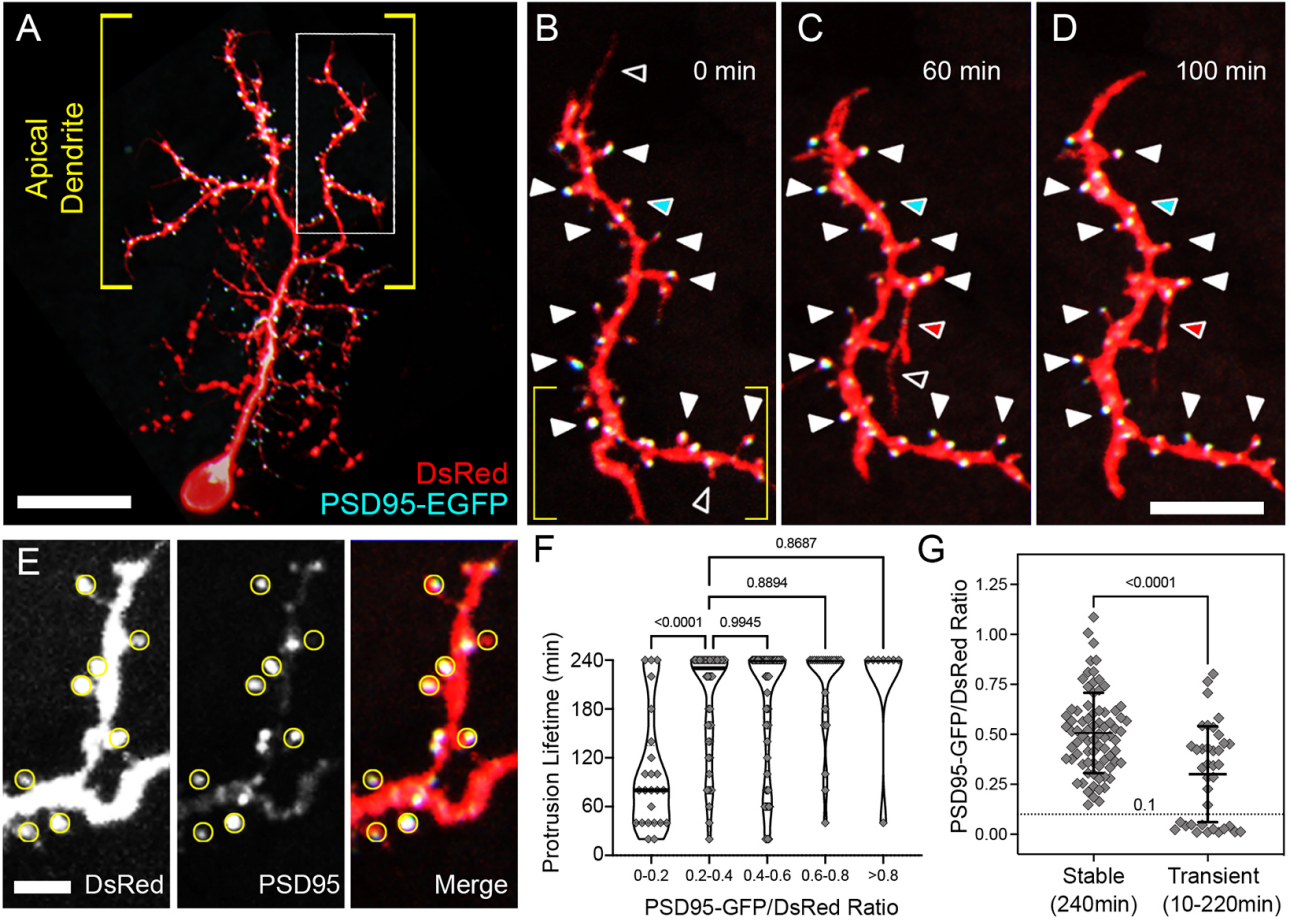
**PSD95-EGFP dynamics in PyrN apical dendrites.** (A) Native orientation view of a 6 dpf PSD95-EGFP/DsRed-labeled PyrN. (B-D) Time-lapse images of PSD95-EGFP localization in a dendrite subregion, as indicated by the boxed region in A. White arrowheads mark seven PSD95-positive spines that were present during the entire 4 h recording. For clarity, some stable spines with PSD95 accumulations are not marked by arrowheads. Open arrowheads indicate PSD95-negative protrusions that were retracted during the time-lapse imaging. Red arrowheads indicate a protrusion that extended, formed a PSD95-EGFP punctum and was stabilized from 60 to 100 min. Cyan arrowheads indicate a spine containing a PSD95 punctum that was retracted while the punctum persisted. (E) Magnified view of subregion indicated by the yellow brackets in B. DsRed (left), PSD95-EGFP (middle) and merged (right) fluorescence channels are shown separately. Yellow circles indicate regions of interest used to calculate PSD95-EGFP/DsRed signal ratios. Note the wide range of PSD95-EGFP intensities within the analysis regions (middle and right panels). (F) Quantification of protrusion lifetimes for spines binned into groups based on their degree of PSD95-EGFP enrichment (PSD95-EGFP/DsRed ratio). Note that there is a wide range of lifetimes for protrusions with the lowest PSD95-EGFP enrichment (ratios from 0-0.2), whereas the majority of protrusions with intermediate (0.2-0.6) or high (>0.6) PSD95-EGFP enrichment values were stable during the 4 h recording. The median for each group is indicated with a horizontal line. One-way ANOVA with Tukey's multiple comparisons test was used to determine significance. Significant differences not shown on graph: 0-0.2 versus 0.4-0.6, *P*<0.0001; 0-0.2 versus 0.6-0.8, *P*<0.0001; 0-0.2 versus >0.8, *P*<0.0001. Analysis was performed on 156 protrusions from five neurons. (G) Comparison of PSD95-EGFP/DsRed ratios between stable and transient protrusions. Note that both groups have many spines with intermediate values, but only the stable group has several spines with ratios >0.8. Conversely, only the transient group contains protrusions with ratios <0.1. Data are shown as mean±s.d. Two-tailed unpaired *t*-test was used to determine significance. Scale bars: 20 µm (A); 8 µm (B-D); 2.5 µm (E).

To directly examine the relationship between spine stability and the formation of synaptic contacts, we imaged PyrNs expressing the postsynaptic marker PSD95-EGFP and DsRed as a cytosolic marker (Niell et al., 2004). As previously reported (Demarco et al., 2021), high levels of PSD95-EGFP expression resulted in neurons with abnormal morphologies. Few neurons exhibited detectable fluorescence signals of PSD95-EGFP and normal PyrN morphologies, which caused us to exclude the majority of labeled neurons. This low yield precluded detailed analysis at multiple days of development. Therefore, we focused on neurons at 6 dpf, an intermediate timepoint when PyrN apical dendrites have a mix of both stable and transient protrusions (Fig. 4E). Consistent with our previous findings (Demarco et al., 2021), PyrNs contained a high density of PSD95 puncta in their apical dendritic arbor (Fig. 5A). Within the apical dendrite, the majority of puncta were located within spine heads (filled arrowheads in Fig. 5B-D). Preliminary examination also revealed that protrusions with PSD95-positive heads tended to be stable (filled arrowheads in Fig. 5B-D), whereas protrusions lacking puncta tended to retract (open arrowheads in Fig. 5B,C). We also observed instances in which nascent protrusions formed a PSD95-EGFP punctum and became stabilized (red arrowhead in Fig. 5C,D), as well as instances in which a spine was retracted but its PSD95 punctum persisted (cyan arrowhead in Fig. 5C,D).

To examine the relationship between PSD95 enrichment and protrusion stability, we measured the fluorescence intensity ratio between PSD95-GFP and DsRed at the tip of each protrusion at the first timepoint of each 4 h time-lapse recording (Fig. 5E). Subsequently, we measured the lifetime of each protrusion to determine whether there was a correlation between PSD95 enrichment and stability. This analysis revealed a statistically significant difference between the stability of protrusions with a low PSD95:DsRed ratio (0-0.2) versus those with higher ratios (*P*≤0.0006 for all pairwise comparisons, one-way ANOVA with Tukey's multiple comparisons test; Fig. 5F). However, there was not a linear relationship between the PSD95:DsRed ratio and protrusion lifetime. For example, protrusions with intermediate PSD95:DsRed ratios (0.2-0.4) had similar average lifetimes as those with higher ratios (0.4-0.6, 0.6-0.8 and ≥0.8; *P*>0.87; Fig. 5F). To further examine this effect, we grouped protrusions by their lifetime (stable or transient) and plotted their corresponding PSD95:DsRed ratios (Fig. 5G). This analysis revealed a highly significant difference in the average PSD95:DsRed ratio for transient versus stable protrusions (*P*<0.0001, unpaired two-tailed *t*-test), in part due to the transient nature of every protrusion with a very low PSD95:DsRed ratio (≤0.1; Fig. 5G). We found that 96.2% (75 of 78) of stable spines with lifetimes of 240 min contained PSD95:DsRed ratios >0.2, although a considerable fraction of transient spines did as well (64.9%, 24 of 37). These findings support a model in which a minimum amount of PSD95 is required for stabilization (≥0.2 in our experiments). However, even relatively high levels of PSD95 enrichment did not necessarily protect spines from retraction. This is likely due to PSD95 being an early marker of postsynaptic contacts. Long-term spine stabilization likely requires recruitment of additional PSD components, such as glutamate receptors and cytoskeletal proteins.

### Impaired apical dendrite growth in *fmr1* mutant larvae

**Fig. 6.**
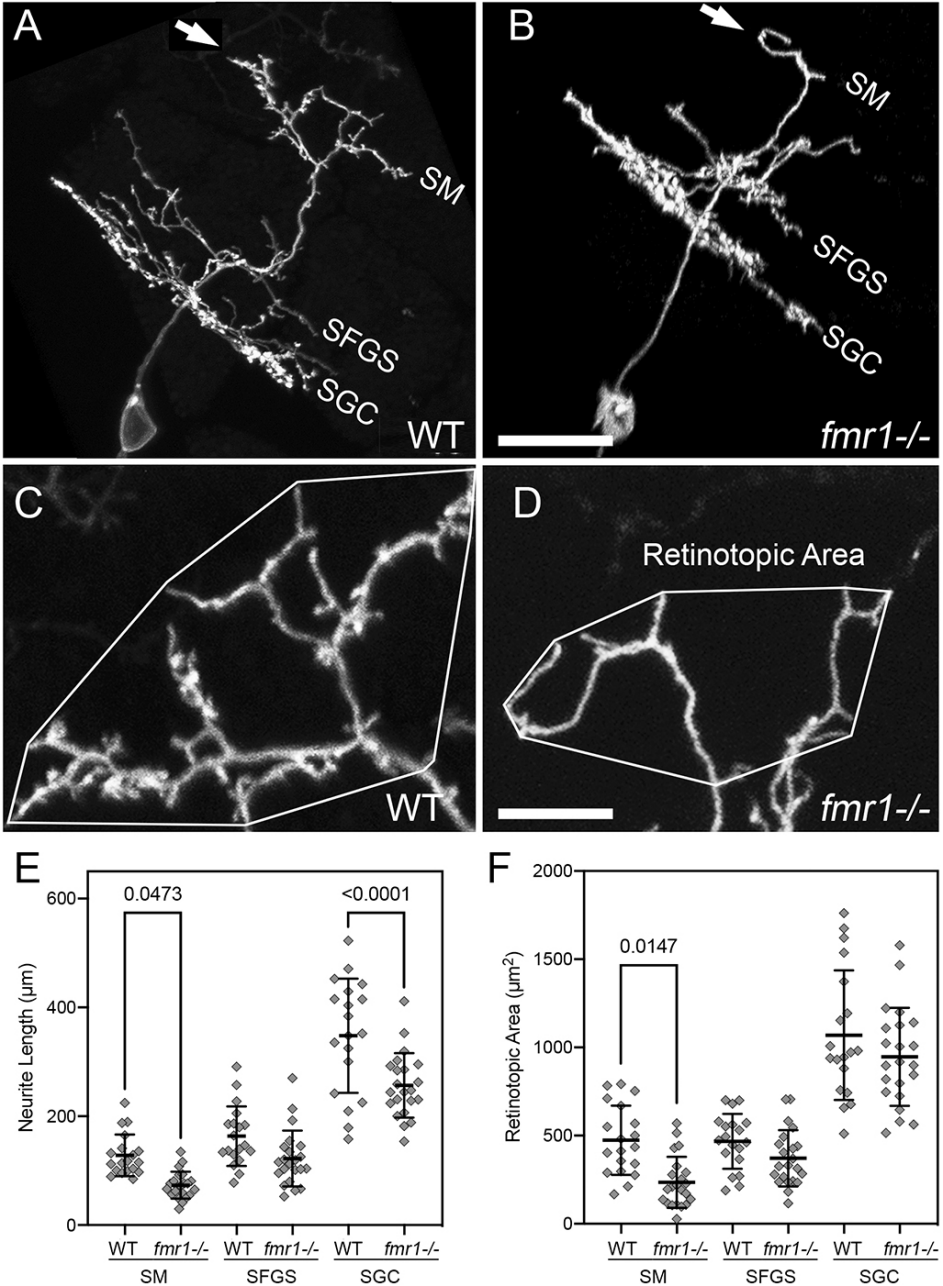
***fmr1* mutants exhibit defects in PyrN dendrite development.** (A,B) Sideview rotated image volumes of a PyrN in a WT 8 dpf larva (A) and an 8 dpf *fmr1*^−/−^ larva. Note three distinct dendrite stratifications in layers SM, SFGS and SGC. Arrows in A and B indicate the apical dendrite. (C,D) Native orientation view (dorsal side up, as in Fig. 1B) of the SM-targeted apical dendrites in the two PyrNs shown in A,B. Convex polygons overlayed on the arbor demonstrate how the retinotopic area was calculated. Note the reduction in dendrite length and area, as well as very few spines on the *fmr1* mutant dendrite. (E) Dendrite arbor-specific neurite length measurements in WT versus *fmr1* mutants. Note significant reductions for the SM and SGC arbor in the mutant. (F) Retinotopic measurements in WT versus *fmr1* mutants. Note the significant reduction in retinotopic area only for the SM dendrite arbor in *fmr1* mutants. Data are shown as mean±s.d. One-way ANOVA with Tukey's multiple comparisons test was used to determine significance. Analysis performed on 19 PyrNs in 16 WT larvae and 22 PyrNs in 19 *fmr1* mutant larvae. Scale bars: 15 µm (A,B); 5 µm (C,D).

To examine dendrite development in *fmr1* mutant larvae, we generated *Tg(id2b:gal4)* fish harboring the *hu2787* mutation of *fmr1* (see Materials and Methods; den Broeder et al., 2009). Embryos generated from incrosses of these fish were injected with *uas:egfp-caax* plasmid DNA to label isolated PyrNs. Live imaging of EGFP-caax-labeled neurons in 8 dpf *fmr1* mutant larvae revealed normal PyrN dendrite stratifications in the SM, SFGS and SGC layers of the tectum (Fig. 6A,B). These image volumes also consistently revealed smaller and less complex apical dendrites in *fmr1* mutant larvae (Fig. 6A,B). To quantify this effect, we used the Simple Neurite Tracer (SNT; Arshadi et al., 2021) plugin for Fiji/ImageJ to semi-automatically generate skeletonized 3D tracings of PyrNs in wild-type (WT) and *fmr1^−/−^* mutants. These tracings were used to measure neurite length in 3D for SM-, SFGS- and SGC-stratified PyrN arbors. Quantification of neurite length measurements revealed significant decreases in both SM and SGC arbors in *fmr1^−/−^* larvae (Fig. 6E). To further examine the effects of the *hu2787 fmr1* mutation, we additionally examined SM dendrite growth in *fmr1^+/−^* heterozygous larvae and determined that partial loss of the *fmr1* gene product also impaired SM dendrite growth (Fig. S2). To quantify the synaptic territory of apical dendrites, we calculated the area of each arbor along the retinotopic axes using maximum projections of image subvolumes containing a single PyrN arbor (Fig. 6C,D; also see Fig. 1B). Retinotopic area measurements revealed a significant decrease only in the SM apical PyrN dendrite in *fmr1^−/−^* mutants (Fig. 6F). Decreased SGC arbor size without a decrease in retinotopic area suggested that the primary effect of *fmr1* loss on this arbor was reduced branching. The SGC arbor contains both axonal and dendritic segments (DeMarco et al., 2019; Demarco et al., 2021) and loss of *fmr1* reduces axon arbor complexity in mouse barrel cortex (Bureau et al., 2008); therefore, this effect might be due to disruption of SGC axon branching. The reductions in neurite length and retinotopic area of SM dendrites indicated that loss of FMRP (encoded by *fmr1*) impairs both dendritic growth and branching of the spiny PyrN apical dendrite. The lack of effect on the SFGS dendrite, which does not form spines, suggests that *fmr1* might play a more critical role in the growth and synaptogenesis of spiny dendrites with a high input density.

### Loss of *fmr1* leads to reduced spine densities and immature spine morphologies

**Fig. 7.**
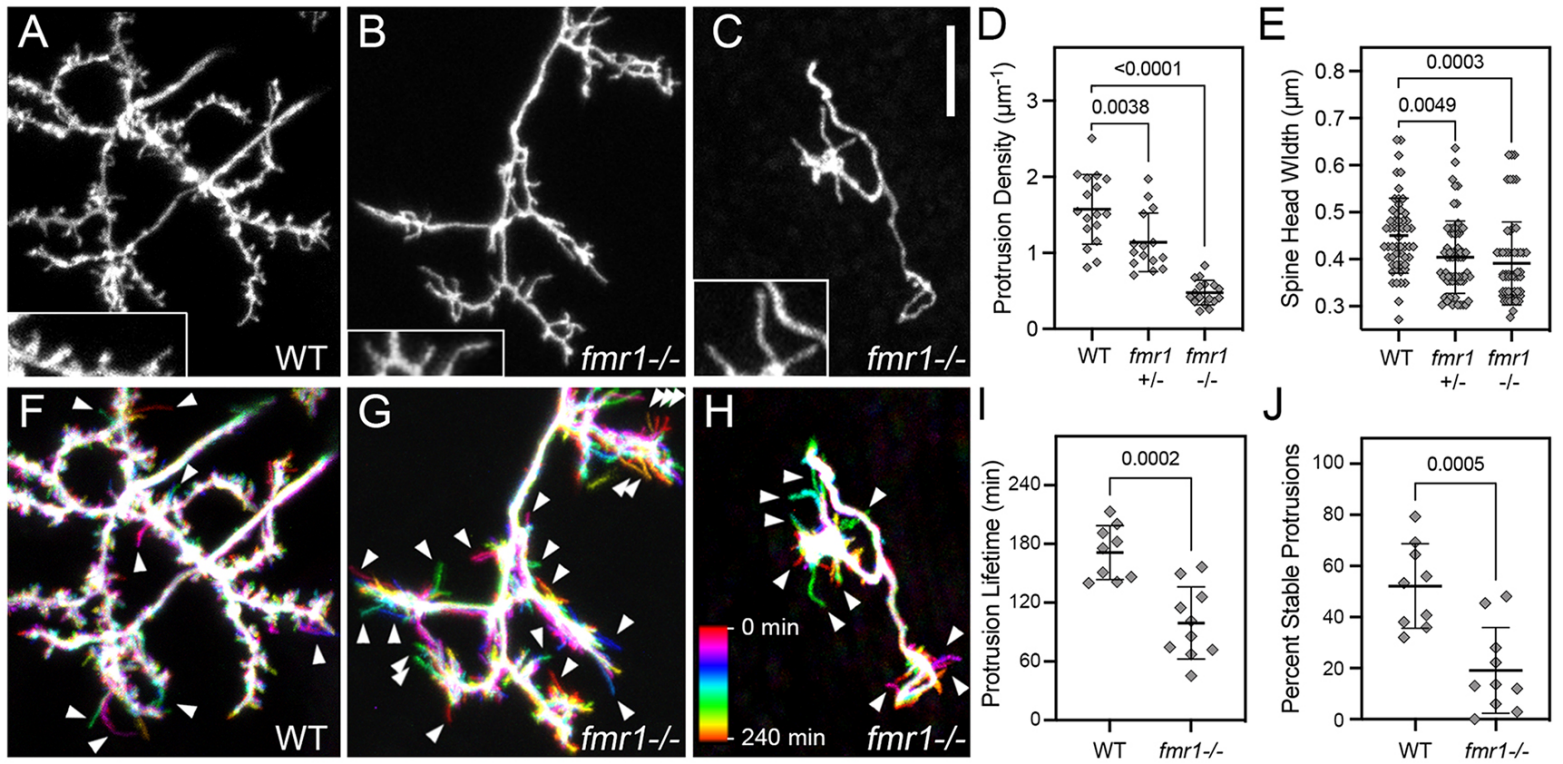
**Reduced spine density, head width and stability in *fmr1* mutants.** (A) Native orientation maximum-projection image (dorsal side up, as in Fig. 1B) of the SM-targeted apical dendrites in an 8 dpf WT larva. (B,C) Native orientation views of the SM-targeted apical dendrites from two 8 dpf *fmr1*^−/−^ mutant larvae. These are representative of mild (B) and severe (C) phenotypes observed in the *fmr1* mutants. Insets in A-C are 1.5× magnified views of subregions containing spines. Note that the WT has both thin and wide heads, whereas the majority of spines on *fmr1*^−/−^ PyrNs have thin heads. (D,E) Quantification of spine density and spine head width for WT, *fmr1* heterozygotes (*fmr1^+/−^*) and *fmr1* homozygotes (*fmr1^−/−^*). Both measurements were made using static single maximum-projection images as those shown in (A-C). Note significant decreases in both density and head width in the *fmr1* mutants. One-way ANOVA with Tukey's multiple comparisons test was used to determine significance. Number of neurons analyzed for protrusion density analysis in WT, *fmr1^+/−^* and *fmr1^−/−^*: 17, 15 and 17, respectively. Number of spines and neurons (indicated in parentheses) analyzed for head width analysis in WT, *fmr1^+/−^* and *fmr1^−/−^*: 55 (4), 65 (6) and 58 (4), respectively. (F-H) Temporal color-coded image of dendrites in A-C during 4 h time-lapse recordings with image volumes acquired every 10 min. Color scale in H applies to all three images and indicates the images corresponding to each timepoint. Note increased number of transient protrusions (single-color label indicating that they were present during a single timepoint) in *fmr1* mutants compared to WT (arrowheads). (I,J) Quantification of the protrusion lifetime and percentage of stable protrusions obtained from 4 h time-lapse recordings with a 10 min acquisition interval. On average, PyrN apical dendrites in *fmr1* mutant larvae had reduced protrusion lifetimes (I), an effect largely due to a significant reduction in the percentage of stable protrusions present for the entire 4 h time-lapse imaging. Data are shown as mean±s.d. *P*-values were obtained using two-tailed unpaired *t*-tests. Analysis was performed on nine PyrNs in nine WT larvae and ten PyrNs in ten *fmr1^−/−^* mutant larvae. Scale bar: 5 µm.

Spine density and morphology were examined in EGFP-caax-labeled PyrNs at 8 dpf, a timepoint when the PyrN apical dendrite contains mostly short spines with enlarged heads (Fig. 3). Structural imaging of WT dendrites revealed a dense array of spines with mature morphologies (Fig. 7A). In contrast, *fmr1^−/−^* mutant dendrites often had a sparse distribution of long, filopodia-like protrusions with narrow heads (Fig. 7B,C; see also Fig. 6D). This trend was observed in mutant dendrites with severely impaired dendrites (Fig. 7C), as well as in those with moderate reductions in dendritic arbor size (Fig. 7B). On average, PyrNs in *fmr1^−/−^* mutants exhibited a greater than threefold reduction in spine density (Fig. 7D; 1.573±0.46 versus 0.477±0.16 µm^−1^, indicated as mean±s.d.; *n*=17 neurons for each condition, *P*<0.0001, unpaired two-tailed Student's *t*-test). PyrNs in *fmr1^+/−^* heterozygous mutants exhibited a moderate reduction in spine density compared to that seen in WT (Fig. 7D). This suggests that PyrNs in *fmr1* mutants were impaired in their ability to form or stabilize spines, as either could lead to a reduction in density. Spine head width was also significantly reduced in PyrNs of both *fmr1* heterozygous and homozygous larvae (Fig. 7E), indicative of more spines with immature morphologies. Developmentally, there was a correlation between spine head width and spine stability (Figs 3 and 4), suggesting that reduced spine density in *fmr1* mutants was caused by a specific deficit in spine stabilization.

### Spine stabilization is impaired in *fmr1* mutant larvae

To determine whether the morphological changes observed in PyrN spines of *fmr1* mutants correlated with changes in spine dynamics, we performed time-lapse imaging of EGFP-caax-labeled PyrNs at 8 dpf. Inspection of these recordings revealed dendrites in *fmr1* homozygous mutants with increased turnover of spines compared to that in WT dendrites (Movie 2). To capture this difference in static images, we generated color-coded temporal projections from these recordings (Fig. 7F-H). WT dendrites formed few motile, filopodia-like protrusions (arrowheads in Fig. 7F), whereas in *fmr1* mutants, protrusions were predominantly motile (arrowheads in Fig. 7G,H). Lifetime analysis confirmed a significant reduction in the average protrusion lifetime in *fmr1* mutants compared to that for WT (Fig. 7I). This was largely due to a marked reduction in the percentage of protrusions with lifetimes of 240 min in *fmr1* mutants (19.3±16.6% in *fmr1* mutants versus 52.3±16.8% in WT, indicated as mean±s.d.; *n*=10 and 9 dendrites, respectively; *P*=0.0005, unpaired two-tailed *t*-test; Fig. 7J). These data support a model in which immature spine morphologies in *fmr1* mutants reflect an inability to form stable synaptic contacts.

## DISCUSSION

Our previous characterization of *id2b:gal4* transgenic larvae provided morphological descriptions of three neuron types labeled in the tectum, as well as their relative proportions (DeMarco et al., 2019). Here, we demonstrate the utility of the *id2b:gal4* transgenic to consistently label PyrNs, a tectal interneuron that forms dendritic spines during larval development. Although these structures are small (typically 1-2 µm in length and less than 0.5 µm in head width), the use of a membrane-targeted EGFP enabled clear, high-resolution imaging of dendritic spines (Fig. 1). Enhanced labeling of spines enabled imaging with reduced laser power using single-photon excitation confocal microscopy. Reduced laser powers facilitated time-lapse imaging for 4 h and, in several instances, repeated time-lapse imaging of the same dendrite at several timepoints throughout development (Fig. 2; Movie 1). Combining these advantages facilitated characterization of spine morphology and motility during early larval development (4-10 dpf). Our findings confirm many similarities between spine development in zebrafish and mammals. Morphologically, spines in mature PyrNs (older than 6 dpf) exhibited a wide range of morphologies, from filopodia-like structures with thin heads to short spines with mushroom heads (Fig. 1). The filopodia-spine transition in PyrNs was characterized by a gradual shift in morphologies, from long filopodia at 4-5 dpf to short spines with enlarged heads at 8-11 dpf (Fig. 3). A similar progression occurs in mammalian pyramidal cell dendrites (Dailey and Smith, 1996; Maletic-Savatic et al., 1999; Ziv and Smith, 1996), although this process occurs more rapidly in zebrafish PyrNs. A unique feature of this system is the ability to examine dendrite branch remodeling in relation to spine development. In mammalian pyramidal cells, these processes are generally not concurrent. In organotypic slices of rat hippocampus, pyramidal cell dendrites go through an early developmental stage (1-2 days in culture) during which transient filopodia predominate and can transform into new dendritic branches (Dailey and Smith, 1996). However, at timepoints at which short, stable spines predominate (1-2 weeks in culture), dendritic arbors do not exhibit changes in branch number or length (Dailey and Smith, 1996). Between 6 and 11 dpf, PyrN dendrites form morphologically mature spines, yet continue to undergo large-scale changes in dendrite morphology (extension, retraction and branching; see Fig. 2). The rapid development of the zebrafish visual system (Niell and Smith, 2005) might drive an accelerated rate of synaptogenesis relative to dendrite development in PyrNs. Alternatively, the dendritic rearrangements during this period might reflect retinotopic refinements necessitated by the continued growth of both retina and brain. We have previously observed changes in the retinotopic position of retinal ganglion cell axon arbors in the tectum during this period of development (Robles et al., 2013). As both the retina and brain continue to grow throughout the life of zebrafish, such rearrangements might persist into adulthood.

PyrN apical dendrites form a dense array of glutamatergic postsynaptic specializations containing PSD95 (Demarco et al., 2021). High-resolution imaging of subcellular PSD95-EGFP localization in PyrN apical dendrites confirmed that the majority of spine heads contain punctate enrichments of PSD95 (Fig. 5). Time-lapse analysis of PSD95-EGFP dynamics allowed us to define the relationship between spine lifetime and PSD95-EGFP accumulation. Our data indicate the vast majority of stable spines contain discrete PSD95 accumulations. Although spines lacking PSD95-EGFP accumulations were generally short-lived, there was only a weak correlation between PSD95-EGFP enrichment and spine stability. This is evidenced by the similar mean lifetimes of spines with high or intermediate PSD95-EGFP/DsRed ratios (Fig. 5). These results are similar to previous findings in mouse cortical pyramidal neurons, in which PSD95 accumulations can be present in both short-lived and stable spines (Cane et al., 2014). Together, these findings support a model in which PSD95 is an early marker of synapses formed by spines, labeling both short-lived and long-lived spines. This early role in synapse formation is likely to involve the well-characterized role of PSD95 in recruiting and clustering AMPA and NMDA-type glutamate receptors (Chen et al., 2015).

To validate the utility of our genetic labeling approach to study neurodevelopmental disorders, we used this approach to characterize spine development in *fmr1* mutants. Our finding that *fmr1* mutants exhibit reductions in spine stability is consistent with previous observations in the mouse cortex (Cruz-Martín et al., 2010; Pan et al., 2010). In both of these studies, FMRP-deficient dendrites contained a higher percentage of transient protrusions compared to WT controls. In mouse layer 5 pyramidal neurons, transient spines were also shown to have reduced spine head widths and volumes (Pan et al., 2010). Although our data revealed a reduction in spine head width for PyrNs in *fmr1* mutant larvae, this effect on spine head width might be cell-type specific. For example, in FMRP-deficient Purkinje cells, spine immaturity is reflected in increased spine lengths without a change in head width (Koekkoek et al., 2005). Unlike our study and others in *Fmr1* KO mice (Comery et al., 1997; Nimchinsky et al., 2001), Purkinje cells also did not exhibit a change in spine density or dendritic arbor complexity. One possible explanation is that FMRP might play a more critical role in circuits in which spiny neurons undergo high levels of activity-dependent synapse refinement. Purkinje dendrite arborization and synaptogenesis might be genetically specified to a greater extent than cortical neurons and tectal PyrNs. If the severity of the phenotype in *fmr1* mutants reflects dependence on activity-dependent competition, this suggests that PyrN dendrite arborization is tightly controlled by activity-dependent mechanisms. Further studies will be required to determine whether spine stabilization in PyrNs is activity dependent.

The *fmr1^hu2787^* mutant is the best characterized ASD-related zebrafish mutant, both behaviorally and physiologically (Constantin et al., 2020; Ng et al., 2013; Shamay-Ramot et al., 2015). The strong defects we observed in dendrite development and spine stabilization might seem surprising considering the mild behavioral defects (Ng et al., 2013) and normal visual responses (Constantin et al., 2020) previously reported in these mutants. One possibility is that only certain types of neurons have a stringent requirement for normal levels of FMRP during development, making them more susceptible to its absence. Tectal PyrNs form part of a reciprocal circuit between the tectum and torus longitudinalis (TL). The apical dendrite in the SM layer of the tectum receives excitatory input from the TL (Folgueira et al., 2020; Northmore, 2017), and our recent anatomical data suggest a high degree of input convergence at the TL-PyrN synapse (Demarco et al., 2021). TL axons providing input to the PyrN apical dendrite in SM form extremely large axonal arbors with a high degree of overlap. These large synaptic territories distribute information from each TL neuron to PyrNs located throughout the tectum. In stark contrast to TL axons, PyrN apical dendrites are small and contain as many as 100 postsynaptic specializations (Demarco et al., 2021). High input densities onto PyrN apical dendrites might necessitate the formation of spines, which increase dendritic surface area and isolate synapses within separate subcellular compartments. We propose that spiny dendrites with high input densities are more susceptible to loss of FMRP than aspiny dendrites. Consistent with this possibility, loss of FMRP had no effect on the morphology of the non-spiny SFGS-targeted PyrN dendrites.

One possible explanation for PyrN sensitivity to loss of FMRP is that a high density of presynaptic terminals requires their apical dendrites to simultaneously sample many different potential partners. Consistent with this, presynaptic TL axons form a dense, mesh-like plexus in the SM layer of the tectum (Demarco et al., 2021) and PyrN protrusion density remains relatively high throughout larval development (Fig. 2K). This might create an increased need for the early components of PSDs, such as scaffolding proteins and glutamate receptors. In the mouse cortex and hippocampus, FMRP binds to mRNAs encoding several components of the PSD, including scaffolding proteins of the Shank, MAGUK and SAPAP families, as well as subunits of NMDA-type and AMPA-type glutamate receptors (Schütt et al., 2009). At the protein level, loss of FMRP reduced levels of the PSD protein SAPAP3, and increased levels of SAPAP1, glutamate ionotropic receptor AMPA type subunit 1 (GRIA1) and glutamate ionotropic receptor NMDA type subunit 1 (GRIN1). However, this study also found cell-type-specific effects on protein levels when comparing cortical and hippocampal neurons. Disruption in the balanced levels of synaptic proteins might disrupt normal synapse assembly and/or disassembly. Synaptotropic models of dendrite growth propose that stable synaptic contact biases the direction of growth by protecting a subset of branches from retraction while others are retracted (Cline and Haas, 2008; Niell, 2006). From this view, the mature dendritic arbor morphology reflects the location of strong connections with appropriate presynaptic partners. Thus, impaired dendrite growth in *fmr1* mutant PyrNs likely arises due to the inability of the neurons to form strong synaptic contacts that protect branches from retraction.

In conclusion, *in vivo* imaging of dendritic spine morphogenesis in larval zebrafish was enabled by a genetic labeling system that targets tectal PyrNs. In addition to confirming similarities between spine morphogenesis in zebrafish larvae and mammals, these findings establish the larval zebrafish as a valuable animal model to study cellular mechanisms underlying spine defects in neurodevelopmental disorders. The ability to conduct automated, high-resolution brain imaging in living zebrafish larvae (Early et al., 2018; Pardo-Martin et al., 2010) will enable future studies to monitor PyrN spine morphology and dynamics at higher throughput. In addition, combining this system with genetic approaches to create mosaic labeling in embryos, such as microsatellite instability-mediated stochastic gene expression (Koole and Tijsterman, 2014) or mosaic analysis with double markers (Xu et al., 2022), holds the promise of eliminating the most laborious step in our current workflow, i.e. early-stage embryo injections. Successful optimization of person-hours required to generate larvae with sparse labeling will enable several scalable screening approaches, such as high-throughput drug screening (MacRae and Peterson, 2015), forward genetic screening (Muto et al., 2005) and reverse genetic screening using CRISPR (Shah et al., 2015). Although we have focused on a zebrafish model of fragile X syndrome, there are currently more than a dozen validated zebrafish lines with validated loss-of-function mutations in ASD risk genes (Rea and Van Raay, 2020). The genetic labeling system we have described will be an important tool in dissecting the effects of these mutations on dendritic spine development.

## MATERIALS AND METHODS

### Fish lines

Zebrafish adults and larvae were maintained at 28°C on a 14/10 h light/dark cycle. Embryos and larvae were raised in 0.3× Danieau’s medium consisting of 17.4 mM NaCl, 0.21 mM KCl, 1.50 mM Hepes buffer, 0.18 mM Ca(NO_3_)_2_ and 0.12 mM MgSO_4_.

*Tg(id2b:Gal4-VP16)mpn215* and *Tg(UAS-E1B:NTR-mCherry)c264* transgenic lines have been previously described (Davison et al., 2007; Förster et al., 2017). All larvae used were either mutants for *mitfa^−/−^* (*nacre*) or double mutants for *mitfa^−/−^* and *roy^−/−^* (*casper*). Use of these pigmentation mutants eliminated the need to chemically block skin pigmentation with phenylthiourea. All animal procedures conformed to the institutional guidelines of the Purdue University Institutional Animal Care and Use Committee. *fmr1^hu2787^* mutant fish (den Broeder et al., 2009) were obtained from the Zebrafish International Resource Center. Equal numbers of male and female adults were used for matings to generate embryos. All larvae were imaged between 4 and 11 dpf, prior to the start of sexual differentiation at 21 dpf. All fish strains, plasmids, chemicals and software are also summarized in Table S1.

### Genotyping

Adult and larval fish were genotyped as described previously (Ng et al., 2013). Briefly, we used the following PCR primers: forward primer, 5′-CTAAATGAAATCGTCACATTAGAGAGGGTA-3′, and reverse primer, 5′-TCCATGACATCCTGCATTAG-3′. PCR products were digested with the RsaI restriction enzyme to identify WT and homozygotes. *fmr1^hu2787^* mutant fish in the A/B strain were mated with *Tg(id2b:gal4,uas:NTRmcherry)* fish in the *mitfa^−/−^*/*roy^−/−^* background. Heterozygotes were identified in the subsequent generation and mated with WT *casper* fish to generate heterozygotes carrying the *id2b:gal4* transgene. These heterozygotes were in-crossed for embryo injections and larvae with sparse labeling were imaged and then genotyped by PCR. Uninjected embryos from these incrosses were also reared to generate adult homozygotes, which were healthy and exhibited normal fecundity. Approximately half of *fmr^−/−^* mutant data in this study were obtained from in-crosses of *fmr^−/−^* adult fish.

### Embryo injections

Genetic mosaic labeling of single neurons by expression of a membrane-targeted EGFP was achieved by injection of the 4xnrUAS:EGFP-caax plasmid (a gift from Bruce Appel and Jacob Hines, University of Colorado, Denver, CO, USA) along with the RNA-encoding Tol2 transposase into WT or mutant embryos transgenic for *id2b:Gal4-VP16.* Labeling of single neurons by co-expression of a PSD95-EGFP fusion protein and DsRed as a cytosolic marker was achieved by injection of the 14UAS PSD95:GFP 5UAS DSRedExpress plasmid (Addgene plasmid #74315). All DNA constructs were pressure-injected at a concentration of 25-50 ng/l into one- to eight-cell-stage embryos.

### Confocal imaging

For live confocal imaging between 4 and 11 dpf, larvae were anesthetized in 0.016% tricaine and embedded in 2% low-melting-point agarose. Imaging was performed on a Nikon C2 confocal microscope equipped with solid state lasers for excitation of EGFP (488 nm) and mCherry/TagRFP (555 nm). Whole-brain imaging of larvae was performed using a Nikon LWD 16×0.8 NA water immersion objective using 1-1.5 µm *z*-steps. Larvae with single-labeled neurons were imaged using a Nikon 60×1.0 NA water immersion objective and 0.375-0.5 µm *z*-steps. For time-lapse recordings, the laser power was lowered to <1% and *z*-stacks with 0.6-1 µm *z*-steps were acquired every 10 min for 4 h, yielding 25 image volumes. For PSD95-EGFP/DsRed imaging, *z*-stacks were acquired every 20 min for 4 h to prevent photodamage due to the increased number of scans per *z*-stack due to two-channel acquisition. Additionally, prior to each time-lapse acquisition, laser power and detector gain for both channels were adjusted to yield images in which spines with bright PSD95-EGFP puncta had PSD95-EGFP/DsRed intensity ratios close to 1.0. This circumvented the need to normalize ratio values when combining data from multiple neurons (Fig. 4F,G). For multi-day imaging, we only used larvae containing single-labeled neurons in one or both tecta. After acquiring the first image volume, the location of the imaged neuron was noted and larvae were released from agarose into a 35 mm Petri dish with 0.3× Danieau’s medium until the next imaging session. For these experiments, we only included data in which a single neuron at the same location was found in each imaging session.

### Image processing and analysis

Image stacks were visualized and analyzed using ImageJ/Fiji software (http://fiji.sc/Fiji). 3D rendering was performed using the 3D Viewer plugin (Schmid et al., 2010). Skeletonized tracings used for calculating neurite lengths were generated with the semi-automated neurite segmentation plugin SNT (Longair et al., 2011). Arbor-specific neurite lengths could be automatically measured in 3D. Retinotopic area was defined as the convex hull area for each arbor when viewed in the native orientation as in Fig. 1A,B (Teeter and Stevens, 2011). Protrusion density was calculated by manually annotating spine locations on maximum projections of apical dendrite image volumes. Protrusion lengths and lifetimes were measured manually in ImageJ/Fiji. Protrusion head widths were measured by obtaining intensity profiles from regions spanning the distal 0.5 µm of each spine as shown in Fig. 3F,H,J. To ensure that bends or swellings of the dendrite were not counted as protrusions, only protrusions with lengths >0.5 µm were analyzed for head width, density or lifetime. Based on our length analysis presented in Fig. 3I, 7.5% of protrusions (79 of 1043) had lengths <0.5 µm. This is the potential undercount due to our length criteria. These criteria were applied uniformly to control and experimental datasets. PSD95-EGFP/DsRed intensity ratios were measured on the first maximum projection of each time-lapse sequence using a circular region of interest with a diameter of 0.5 µm. All data are presented as mean±s.d., except for violin plots, in which the horizontal line indicates the median value.

### Statistical analysis

Raw data acquired in this study are provided in Datasets 1-7. Datasets were analyzed using GraphPad Prism software version 9.2.0 for Mac (GraphPad Software, La Jolla, CA, USA). All data displayed a normal distribution. One-way ANOVA was used to identify differences among means for datasets with three or more groups, with Tukey's multiple comparisons test used for comparisons between groups. For datasets with two groups, comparisons between groups used unpaired two-tailed *t*-tests. *P*-values less than 0.05 were considered significant.

## Supplementary Material

Supplementary information
