# Supplementary material for: A genetic labeling system to study dendritic spine development in zebrafish models of neurodevelopmental disorders
Source: Dis Model Mech. 2022 Aug 19;15(8):dmm049507. doi: 10.1242/dmm.049507 (PMC9403749; doi:10.1242/dmm.049507)
Supplement: Supplementary information [file dmm-15-049507-s1.pdf]

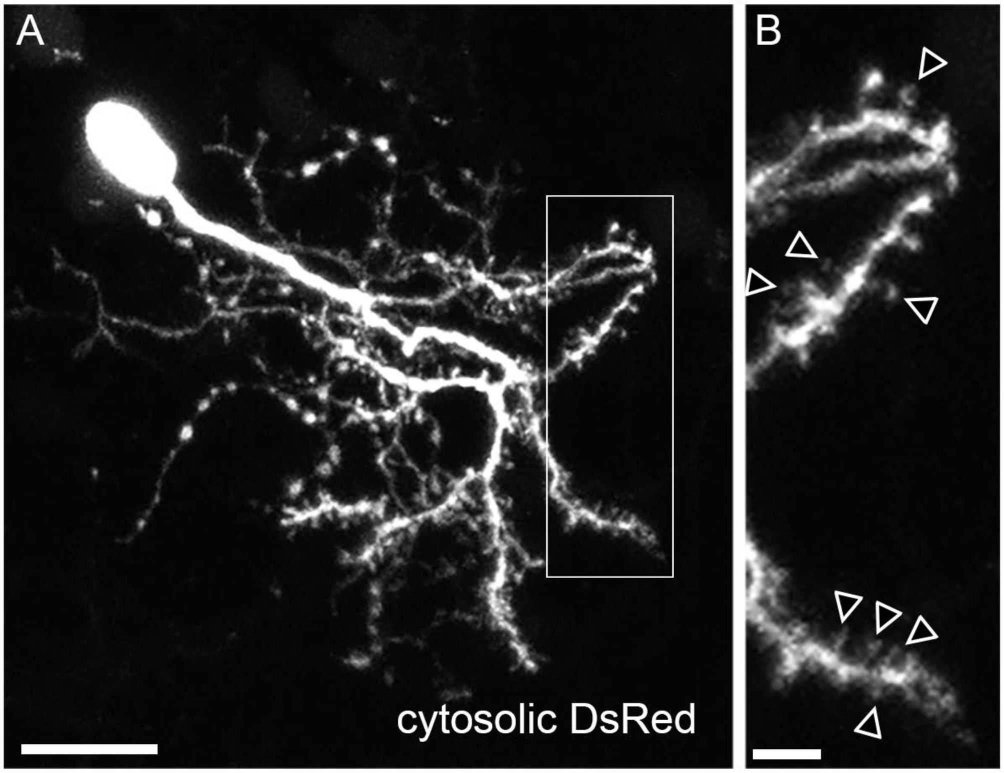

Fig. S1. Maximum projection image of a 6 dpf PyrN labeled by expression of cytosolic DsRed. Open arrowheads indicate putative spines that are dimly labeled, likely due to small head volumes. Scale bars: 10 µm in A, 2.5µm in B.

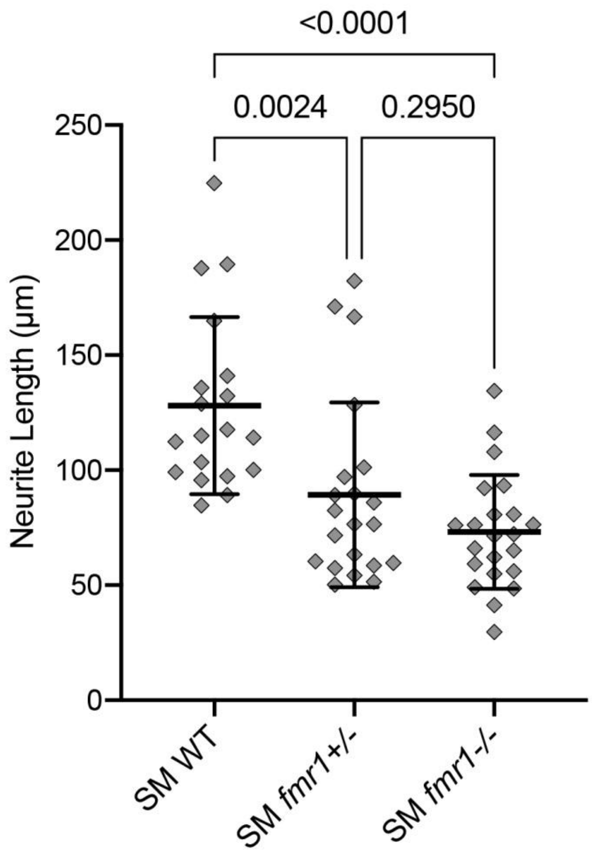

Fig. S2. SM dendrite neurite length measurements in WT, *fmr1* heterozygous and *fmr1* homozygous larvae. Note significant reductions for the SM dendrite in both heterozygous and homozygous mutants. One-way ANOVA with Tukey's multiple comparisons posthoc test. Analysis performed on 19 PyrNs in 16 WT larvae, 21 PyrNs in 16 *fmr1* heterozygous larvae, and 22 PyrNs in 19 *fmr1* homozygous larvae.

Disease Models & Mechanisms • Supplementary information

Table S1. Reagents and resources used in this study

**Experimental Models: Organisms/Strains**		
Tg(id2b:Gal4-VP16)mpn215	Förster et al., 2017	N/A
Tg(UAS-E1B:NTR-mCherry)c264	Davison et al., 2007	ZFIN Cat #: ZL1473
**DNA Plasmids**		
pEXPR-Tol2-4xnrUAS:EGFP-CaaX	Hines et al., 2015	N/A
**Chemicals**		
Tricaine	SigmaAldrich	E10521
UltraPure Low Melting Point Agarose	Invitrogen	16520050
**Software**		
GraphPad Prism	www.graphpad.com/	RRID:SCR_002798
Fiji 1.0	http://fiji.sc	RRID:SCR_002285

Davison, J. M., Akitake, C. M., Goll, M. G., Rhee, J. M., Gosse, N., Baier, H., Halpern, M. E., Leach, S. D. and Parsons, M. J. (2007). Transactivation from Gal4-VP16 transgenic insertions for tissue-specific cell labeling and ablation in zebrafish. *Dev. Biol.* **304**, 811–824.

Förster, D., Arnold-Ammer, I., Laurell, E., Barker, A. J., Fernandes, A. M., Finger-Baier, K., Filosa, A., Helmbrecht, T. O., Kölsch, Y., Kühn, E., et al. (2017). Genetic targeting and anatomical registration of neuronal populations in the zebrafish brain with a new set of BAC transgenic tools. *Scientific Reports* **7**, 5230.

Hines, J. H., Ravanelli, A. M., Schwindt, R., Scott, E. K. and Appel, B. (2015). Neuronal activity biases axon selection for myelination in vivo. *Nat Neurosci* **18**, 683–689.

Disease Models & Mechanisms • Supplementary information

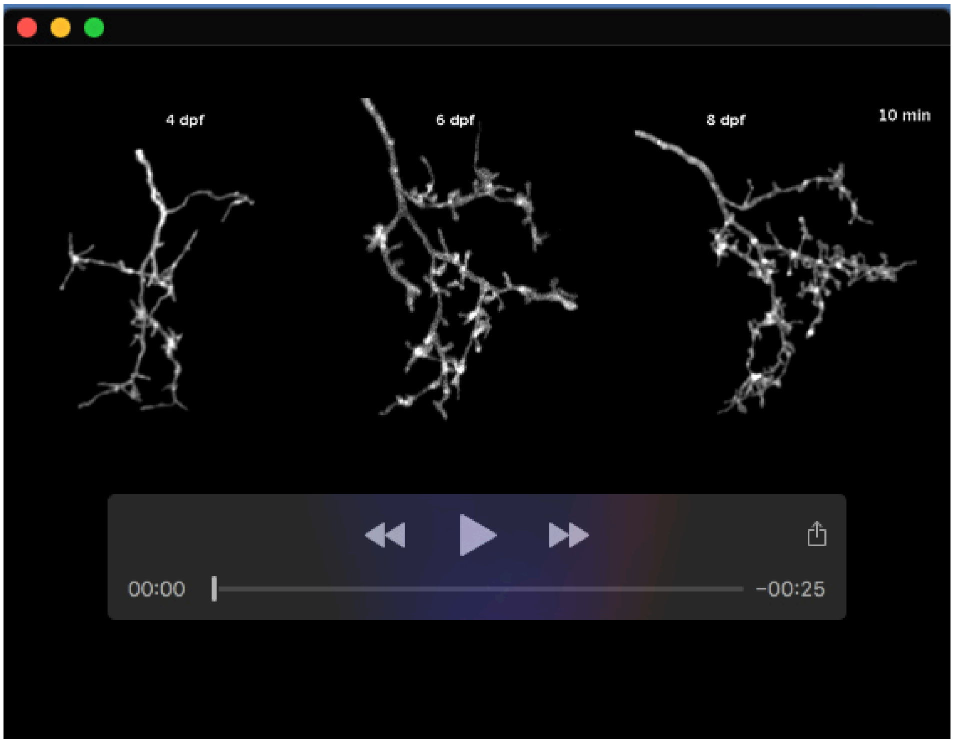

Movie 1. The filopodia-spine transition revealed by live imaging of dendrite protrusion dynamics. Timelapse recordings of the same EGFP-caax labeled PyrN apical dendrite imaged at 4, 6, and 8 dpf. Image volumes were acquired every 10 min for 4 hrs. Note prevalence of long, motile filopodia at 4 dpf, whereas short spines predominate at 8 dpf. At 6 dpf the dendrite contains a mix of long and short protrusions.

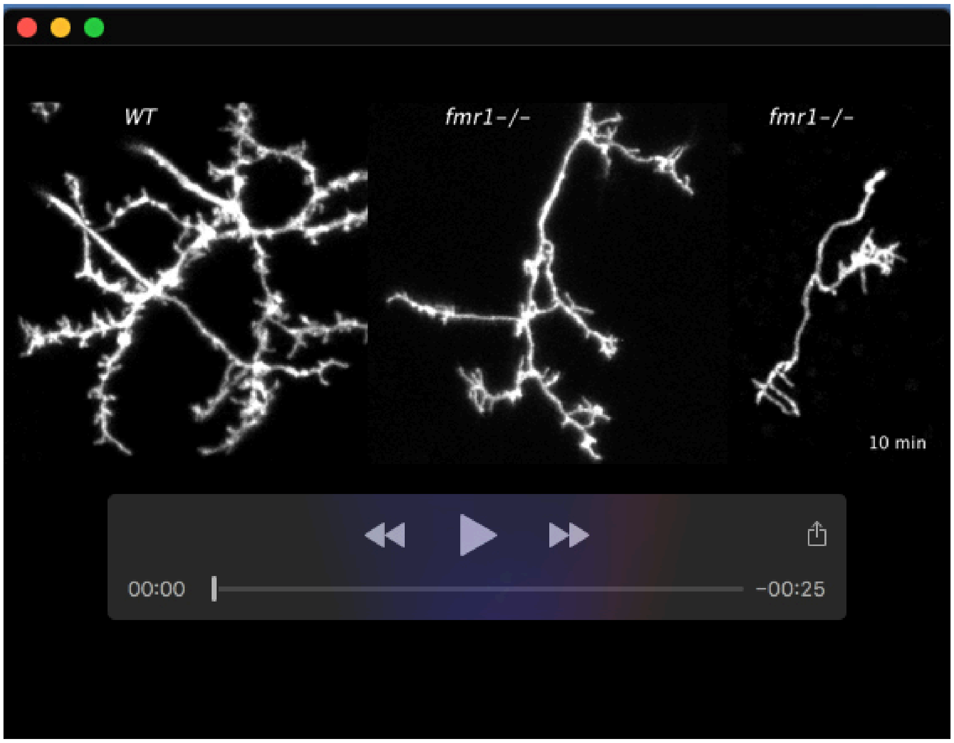

Movie 2. Reduced protrusion stability in *fmr1* mutant PyrNs. Timelapse recordings of the three EGFP-caax labeled PyrN apical dendrites from one WT (left) and two *fmr1* mutant (middle and right) larvae. *fmr1* mutant PyrN at middle exhibited a moderate reduction in dendrite arbor size, whereas *fmr1* mutant PyrN at right exhibited severely impaired dendrite growth. Compare short and stable spines on WT to the motile filopodia present on the mutant PyrNs. Asterisk indicates a 1 hr interval when part of the middle dendrite was below the image acquisition volume due to specimen drift.

Dataset 1. Morphological analysis of PyrN SM dendrites between 4 and 10/11 dpf. For each arbor is provided: filename, area (μm^2), # of spines, neurite length (μm), # of branchpoints, and spine density. Related to Figure 2.

Click here to download Dataset 1

Dataset 2. Protrusion length analysis of PyrN SM dendrites between 4 and 10/11 dpf. For each arbor is provided: length (μm) of each protrusion. Column subdivisions represent measurements obtained from different PyrNs. Related to Figure 3.

Click here to download Dataset 2

Dataset 3. Protrusion head width analysis at 5 and 8 dpf for WT and 8dpf only for Fmr1 heterozygotes and homozygotes. Column subdivisions represent measurements from different PyrNs. Related to Figures 3 and 7.

Click here to download Dataset 3

Dataset 4. Protrusion lifetime analysis between 4 and 10/11 dpf. Column subdivisions represent measurements from different PyrNs. Related to Figure 4.

Click here to download Dataset 4

Dataset 5. Protrusion lifetime analysis measured within bins defined by the PSD95-EGFP/DsRed ratio at the tip of the first frame of the timelapse series. Column subdivisions represent measurements from different PyrNs. Related to Figure 5.

Click here to download Dataset 5

Disease Models & Mechanisms • Supplementary information

Dataset 6. Morphological analysis of 8 dpf PyrN SM dendrites for WT larvae and *fmr1* homozygotes. For each arbor is provided: area (μm2), and neurite length (μm). Related to Figure 6.

Click here to download Dataset 6

Dataset 7. Morphological analysis of 8 dpf PyrN SM dendrites for WT larvae and *fmr1* homozygotes. For each arbor is provided: area (μm2), and neurite length (μm). Related to Figure 7.

Click here to download Dataset 7

Disease Models & Mechanisms • Supplementary information
